# A nomogram for predicting poor sleep quality in patients with systemic lupus erythematosus

**DOI:** 10.3389/fneur.2025.1562949

**Published:** 2025-05-21

**Authors:** Ling Ma, Yan-Hong Li, Xin Guo, Ying Wang, Yin-Lan Wu, Chun-Yu Tan

**Affiliations:** Department of Rheumatology and Immunology, West China Hospital, Sichuan University, Chengdu, China

**Keywords:** systemic lupus erythematosus, poor sleep quality, risk prediction model, clinical prediction, nomogram

## Abstract

**Objective:**

To construct a nomogram for poor sleep quality in patients with systemic lupus erythematosus (SLE).

**Methods:**

Clinical data from 218 SLE patients who visited a tertiary hospital’s rheumatology and immunology department in Chengdu, Sichuan Province, China, between 2021 and 2022 were analyzed. LASSO analysis and multivariate logistic regression were used to identify independent risk factors, and a nomogram was used to integrate and model the various risk factors. The model was evaluated using receiver operating characteristic curves, calibration curves, and decision curve analysis (DCA). Internal validation was conducted using the bootstrap method, and the clinical impact curve (CIC) was used to assess the clinical effectiveness of the predictive model.

**Results:**

In total, 104 patients (47.7%) had poor sleep quality, while 114 patients did not have poor sleep quality (52.3%). The nomogram for predicting poor sleep quality in patients had an area under the curve of 0.789, a sensitivity of 51.92%, and a specificity of 93.86%. The calibration curve closely approximated the ideal curve; DCA showed a threshold probability of 35%; the C-index was 0.789; and the CIC showed a threshold probability of 60%. These results indicate that the nomogram has good predictive accuracy and clinical utility.

**Conclusion:**

We constructed and validated a nomogram for poor sleep quality in patients with SLE, providing a convenient and reliable tool for the clinical prediction of poor sleep quality in these patients. Further multicenter studies are warranted to validate these findings and further elucidate the underlying mechanisms of sleep disturbances in SLE.

## 1 Introduction

Systemic lupus erythematosus (SLE) is a common autoimmune disease characterized by multiorgan involvement, relapses, and remissions (1). Previous studies have shown that the prevalence of SLE in China is 30–70 per 100,000 people (2). With the continuous improvement in the diagnosis and treatment of SLE, the 5-year survival rate of SLE patients worldwide has exceeded 90% (3). Thus, in addition to pharmaceutical treatment, the comprehensive management of patients throughout the disease trajectory and the improvement of their quality of life have become important focuses of patient care (4). In addition, research has shown that sleep quality is severely impaired in SLE patients (5, 6), and more effective intervention requires an understanding of the occurrence and risk factors ofsleep disorders in patients with SLE. Currently, most studies of sleep disorders in patients with SLE focus on the risk factors for SLE-related sleep disorders (7, 8), with different studies focusing on different risk factors and populations. One study based on multiple patient self-reported outcome measures showed that even 62.9% of SLE patients reported poor sleep quality (9). SLE patients with comorbid poor sleep quality experience higher levels of fatigue, emotional distress, and stress, and their quality of life is also compromised (10, 11).

Poor sleep quality also leads to increased disease activity in SLE, promoting disease exacerbation (12, 13). Several studies have reported that more than 50% of patients with SLE experience sleep problems, which are related to disease activity, pain, fatigue, and depression (14). Therefore, early identification and intervention of sleep in SLE patients is a key focus in improving their quality of life.

Using machine learning methods to develop a clinical visualization tool for predicting the risk and patterns of poor sleep quality in SLE patients can help reduce the risk of developing poor sleep quality by addressing the underlying risk factors, and the prediction can be used to plan lifestyle or treatment decisions based on the risk of health conditions at specific periods (7). Therefore, defining an appropriate risk threshold to recommend interventions is a key challenge in translating risk prediction models into clinical practice. However, there is a lack of research on nomograms for predicting the risk of poor sleep quality in SLE patients internationally. Previous studies have shown that nomograms have good clinical application prospects (15–17), but there is currently no such tool for sleep quality assessment in SLE. Early identification and as a part of a comprehensive education on best evidence for patients may lead to better disease control and reduced healthcare utilization in treating SLE.

Therefore, this study included 218 SLE patients, analyzed their demographic and clinical characteristics, fatigue, depression, and other data, and used LASSO and to select independent risk factors and construct and validate a nomogram for poor sleep quality in patients with SLE. This model could provide a reference for clinical practice. This study aimed to evaluate sleep quality and construct a nomogram for poor sleep quality in patients with SLE with the goal of carrying out risk stratification, thereby ensuring that high-risk patients can receive the appropriate care. This results can provide evidence to design targeted and intervention for improving SLE patients’ sleep quality.

## 2 Methods

### 2.1 Study participants

**Figure 1:**
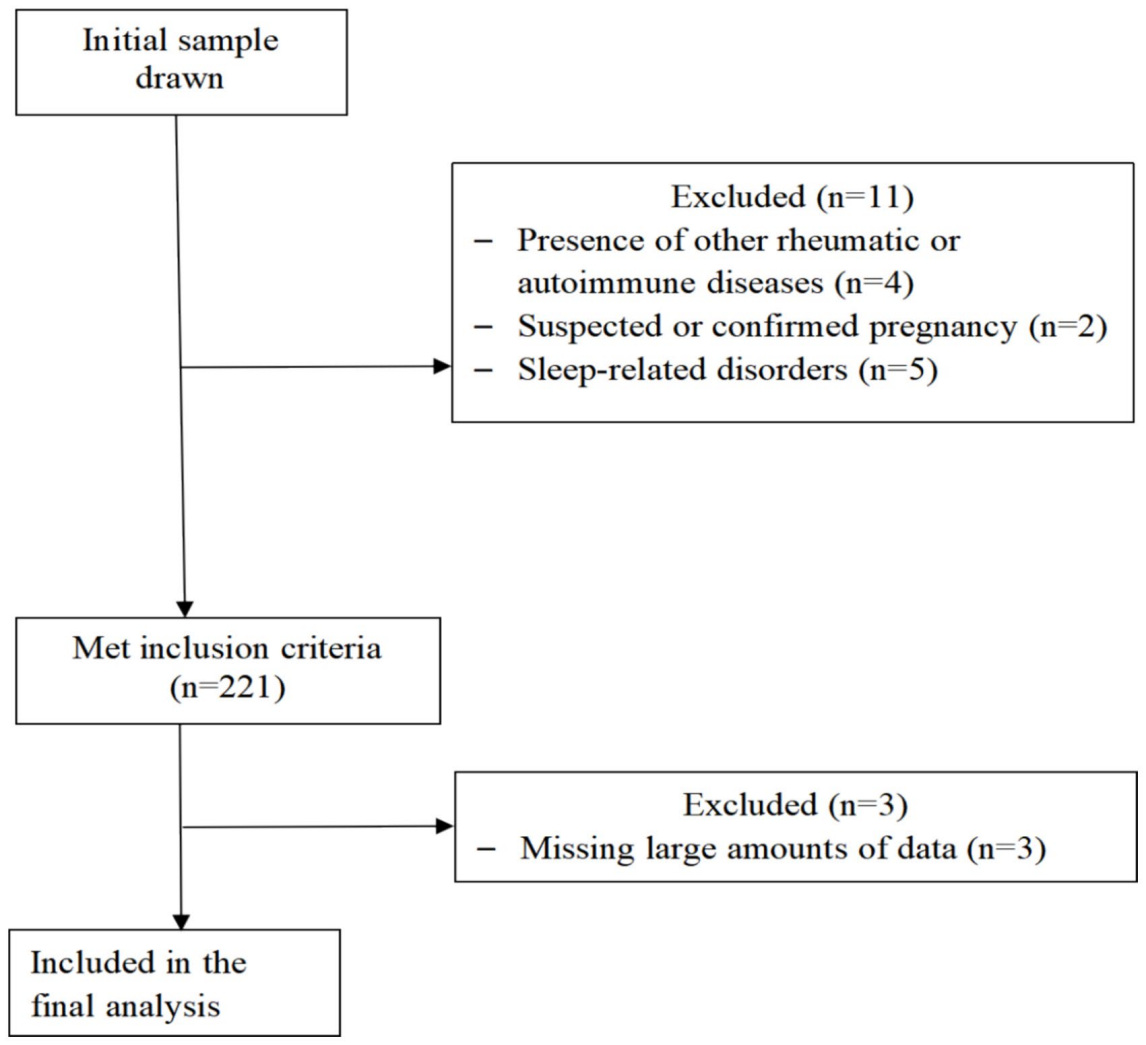
STROBE participant flow diagram. Flow diagram. The flowchart outlines the selection criteria and patient exclusions for the study on sleep quality in patients with systemic lupus erythematosus. This study that included SLE patients recruited from a tertiary hospitals rheumatology and immunology department between 2021 and 2022. Four patients were excluded due to presence of other rheumatic or autoimmune diseases, two suspected or confirmed pregnancy patients, five patients sleep-related disorders and current use of sleeping aids, 221 patients met inclusion criteria, excluding three patients due to incomplete records, the study included valid clinical data from 218 patients.

This was a cross-sectional study that included SLE patients recruited from a tertiary hospital’s rheumatology and immunology department between 2021 and 2022. The study included valid clinical data from 218 patients. The collected data underwent preprocessing, and have complete inspection data, specifically we deleted indicators missing more than 10% of values, and then deleted patients with more than 10% of missing items. The inclusion criteria were as follows: (a) fulfillment of the 1997 American College of Rheumatology and/or the 2012 Systemic Lupus International Collaborating Clinics classification criteria for SLE (8, 18); (b) age between 18 and 70 years, irrespective of gender; and (c) ability to understand the study and provide informed consent. The exclusion criteria were as follows: (a) presence of other rheumatic or autoimmune diseases [such as rheumatoid arthritis, Sjögren’s syndrome, ankylosing spondylitis, scleroderma, dermatomyositis, fibromyalgia (19)]; (b) comorbid significant organ failure or malignancy; (c) severe cognitive impairment, dementia, mental illness, or other neurodegenerative diseases; (d) suspected or confirmed pregnancy; (e) sleep-related disorders and current use of sleeping aids; (f) inability to complete the questionnaire independently or with assistance. The flowchart of patient selection is shown in Figure 1.

This study was approved by the Ethics Committee on Biomedical. Research at the West China Hospital of Sichuan University; and complied with the Declaration of Helsinki.

### 2.2 Data collection

#### 2.2.1 Risk factor selection

See Table 1.

**Table 1:** Risk factor selection and measurement methods.

Variable	Measurement tool	Description
Patient Information	Face-to-face questionnaire	Includes: gender, age, height, weight, body mass index, smoking history, alcohol consumption history, educational level, marital status, occupation, place of residence, family size, average monthly income, annual medical expenses for SLE, duration of disease, medical payment method
SLE Disease Characteristics	Medical records and patient complaints	Includes facial malar rash, headache, nausea/vomiting, arthralgia, diarrhea, fatigue, Raynaud’s phenomenon, vasculitis, cough, chest tightness/chest pain, fever, lower limb edema
SLE Disease Activity	SLEDAI-2000 and SLE-DAS	**SLEDAI-2000**: 24 items covering 9 organ systems, disease activity score range (0–105, higher = more active disease) (55) **SLE-DAS**: 17 items, includes all disease manifestations of the 24, items above, as well as additional items about hemolytic anemia and the involvement of the cardiovascular, pulmonary, and gastrointestinal systems,with higher scores indicating greater disease activity (56)
Anxiety and Depression	Hospital Anxiety and Depression Scale (HADS)	Fourteen items, divided into two subscales: **HADS-A (Anxiety)**: 0–7 = normal, 8–10 = mild, 11–14 = moderate, 15–21 = severe **HADS-D (Depression)**: Same scoring as HADS-A (57)
Fatigue Level	Fatigue Severity Scale (FSS)	Average score of 9 items, ranging from 1.0 (no fatigue) to 7.0 (maximum fatigue). A total score ≥36 points was used as the assessment criterion for fatigue (58, 59)
Red Blood Cell Count (RBC) and Hemoglobin Count (HGB)	Patients’ medical records	Laboratory test results
Smoking History	Self-reported	Previous cigarette smoking and current smoking
Alcohol History	Self-reported	Drinking alcohol ≥1 time per week in the past year (60)
Immunosuppressants and Hormone Use	Medical records	Recorded medication usage.

#### 2.2.2 Outcome measures

The main outcome measure of this study was the presence of poor sleep quality. The Pittsburgh Sleep Quality Index (PSQI) was used for the assessment. The PSQI is a self-reported questionnaire developed by Dr. Buysse and colleagues from the Department of Psychiatry at the University of Pittsburgh in 1989 that is used to evaluate sleep quality and disturbances over a one-month interval. The questionnaire consists of 18 questions that can be transformed into seven components and a global score. The seven components are subjective sleep quality, sleep latency, sleep duration, habitual sleep efficiency, sleep disturbances, use of sleep medication, and daytime dysfunction. The global score is the sum of the seven component scores. The total score ranges from 0 to 21, with higher scores indicating poorer sleep quality. Since its development, the PSQI has been widely used to measure sleep quality in different patient populations (20). In the present study, a score ≥ 7 was considered indicative of poor sleep quality, while a score < 7 was considered indicative of no poor sleep quality (21).

The normal range for RBC was 3.8–5.1 × 10^12^/L and the normal range for HGB was 115–150 g/L.

### 2.3 Statistical methods

#### 2.3.1 Univariate analysis

Data analysis was performed using R software (version 4.2.1). Count data were described as n (%), and quantitative data were tested for normality using the Shapiro–Wilk test. Data that followed a normal or approximately normal distribution were presented as means with standard deviation (SD), while non-normally distributed quantitative data were described using medians (P25, P75). For comparisons between two groups, independent-sample *t* tests were used for normally distributed data, and Mann–Whitney *U* tests were used for non-normally distributed data. Count data were analyzed using chi-square tests.

#### 2.3.2 Construction of a column chart

We used the least absolute shrinkage and selection operator (LASSO) to identify the predictors of poor sleep quality. LASSO (22) minimizes the sum of squares of the residuals between the absolute values of the regression coefficients and with less than one constant, it produces a regression coefficient that is strictly equal to 0. Then, we obtained an interpretable model, selected the independent variables that had the greatest impact on the dependent variables, and calculated the corresponding regression coefficients. Due to the excellent performance of this method in variable screening and model stability, many researchers in the medical field have used the LASSO method to build models for prediction (23). The variables selected by the LASSO regression analysis were used as the independent variables in a binary logistic regression analysis to fit a multivariate logistic regression model and determine the independent risk factors for poor sleep quality. The independent risk factors determined by the binary logistic regression analysis were used to construct a column chart prediction model for sleep disorder risk (24, 25). This model allowed for the intuitive calculation of the risk of poor sleep quality based on patient information.

#### 2.3.3 Model validation

Internal validation was performed using Bootstrap resampling with 2000 iterations. Poor sleep quality was used as the dependent variable, and the total score from the column chart risk assessment model was used as the independent variable. Goodness-of-fit tests and likelihood ratio tests were performed, and the predictive accuracy was described using the area under the receiver operating characteristic (ROC) curve (AUC). An ROC curve was plotted, and odds ratios (ORs) with 95% confidence intervals (CIs) were calculated to validate the reliability of the model (26). A significance level of *p* < 0.05 was used for all tests.

#### 2.3.4 Sample size calculation

Sample size calculation was based on the logistic regression prediction model. It is generally recommended to have at least 10 events per variable (EPV) to eliminate coefficient bias (27). Assuming that 10 outcome events were selected based on the LASSO regression results, a minimum of 10 × 10 = 100 cases were required. Considering that the main end point of this study was the poor sleep quality assessed by the PSQI score, and based on the previous research indicating a sleep disorder prevalence of 62.9% in SLE patients, at least 100/0.629 ≈ 159 cases were needed. Additionally, considering the potential loss to follow-up, an additional 20% was added, resulting in a minimum requirement of 191 SLE patients to meet the sample size requirement for constructing the prediction model.

## 3 Results

### 3.1 General characteristics of the study population

In total, 218 SLE patients were included, namely 23 males and 195 females. The mean age of the patients was 35.2 ± 11.4 years, and the mean disease duration was 71.84 ± 82.70 months. The occurrence rate of poor sleep quality in SLE was 47.7%. Comparative analysis between the group with and without poor sleep quality revealed statistically significant differences in age, gender distribution, disease duration, educational level, and history of alcohol consumption (*p* < 0.05; Table 2).

**Table 2:** General characteristics of the study population.

Variable	Category	Poor sleep quality (*n* = 104)	No poor sleep quality (*n* = 114)	t/Z/χ^2^	*p* value
Age (years)		37.2 ± 12.0	33.4 ± 10.7	−2.320^a^	0.015
Gender	Male	5 (4.8%)	18 (15.8%)	6.950^c^	0.008
	Female	99 (95.2%)	96 (84.2%)		
Disease duration (months)		36(7,108)	51.5(10,120)	−2.177^b^	0.031
BMI		21.71 ± 3.19	21.31 ± 2.94	−0.990^a^	0.331
Education	Primary school or below	8 (7.7%)	8 (7%)	−2.80^b^	0.005
	Middle school	27 (26%)	14 (12.3%)		
	High school or technical school	20 (19.2%)	16 (14%)		
	Junior college or above	49 (47.1%)	76 (66.7%)		
Marital status	No spouse	34 (32.7%)	46 (40.4%)	1.373^c^	0.242
	Married	70 (67.3%)	68 (59.6%)		
Occupation	Employed	50 (48.1%)	61 (53.5%)	2.902^c^	0.407
	Student	11 (10.6%)	16 (14%)		
	Farming or self-employed	12 (11.5%)	14 (12.3%)		
	Unemployed or retired	31 (29.8%)	23 (20.2%)		
Smoking history	No	102 (98.1%)	106 (93.0%)	3.225^c^	0.105
	Yes	2 (1.9%)	8 (7.0%)		
History of alcohol consumption	No	99 (95.2%)	96 (84.2%)	6.950^a^	0.008
	Yes	5 (4.8%)	18 (15.8%)		
Place of residence	Urban	92 (88.5%)	98 (86%)	0.303^c^	0.583
	Rural	12 (11.5%)	16 (14%)		
Monthly family income (Yuan/month)	< 1,000	7 (6.7%)	8 (7%)	−0.914^c^	0.361
	1,000–1999	12 (11.5%)	6 (5.3%)		
	4,000–5,999	25 (24%)	32 (28.1%)		
	6,000–7,999	8 (7.7%)	7 (6.1%)		
	8,000–9,999	9 (8.7%)	7 (6.1%)		
	10,000–14,999	12 (11.5%)	10 (8.8%)		
	15,000–19,999	3 (2.9%)	7 (6.1%)		
	≥ 20,000	3 (2.9%)	8 (7%)		
SLE annual medical expenses (Yuan/year)	< 1,000	5 (4.8%)	12 (10.5%)	−1.64^b^	0.101
	1,000–1999	4 (3.8%)	6 (5.3%)		
	2000–3,999	10 (9.6%)	12 (10.5%)		
	4,000–5,999	6 (5.8%)	4 (3.5%)		
	6,000–7,999	4 (3.8%)	6 (5.3%)		
	8,000–9,999	4 (3.8%)	5 (4.4%)		
	10,000–14,999	17 (16.3%)	24 (21.1%)		
	15,000–19,999	12 (11.5%)	7 (6.1%)		
	≥ 20,000	42 (40.4%)	38 (33.3%)		

a Stands for the use of independent-sample *t* tests.

b Stands for the use of Mann–Whitney U tests.

c Stands for the use of the chi-square test.

### 3.2 Disease characteristics of SLE patients

Comparative analysis between the group with and without poor sleep quality revealed statistically significant differences in anxiety and depression status, fatigue, RBC, and HGB levels (*p* < 0.05; Table 3).

**Table 3:** Disease characteristics of the study population.

Disease characteristic	Characteristic category	Poor sleep quality (*n* = 104)	No poor sleep quality (*n* = 114)	t/Z/χ^2^	*p* value
PSQIscore					
Anxiety score	No anxiety	84 (80.8%)	108 (94.7%)	−3.207^c^	0.001
	Possible anxiety	13 (12.5%)	5 (4.4%)		
	Moderate to severe anxiety	7 (6.7%)	1 (0.9%)		
Depression score	No depression	83 (79.8%)	108 (94.7%)	−3.357^c^	0.001
	Possible depression	15 (14.4%)	5 (4.4%)		
	Moderate to severe depression	6 (5.8%)	1 (0.9%)		
Fatigue	No fatigue	55 (52.9%)	94 (82.5%)	21.984^c^	<0.001
	Fatigue	49 (47.1%)	20 (17.5%)		
RBC	Decreased	21 (20.2%)	6 (5.3%)	−2.268^c^	0.023
	Normal	78 (75%)	107 (93.9%)		
	Increased	5 (4.8%)	1 (0.9%)		
HGB	Decreased	18 (17.3%)	6 (5.3%)	−2.570^c^	0.010
	Normal	85 (81.7%)	108 (94.7%)		
	Increased	1 (1%)	0 (0%)		

a Stands for the use of independent-sample t tests.

b Stands for the use of Mann–Whitney U tests.

c Stands for the use of the chi-square test.

### 3.3 LASSO regression analysis

**Figure 2:**
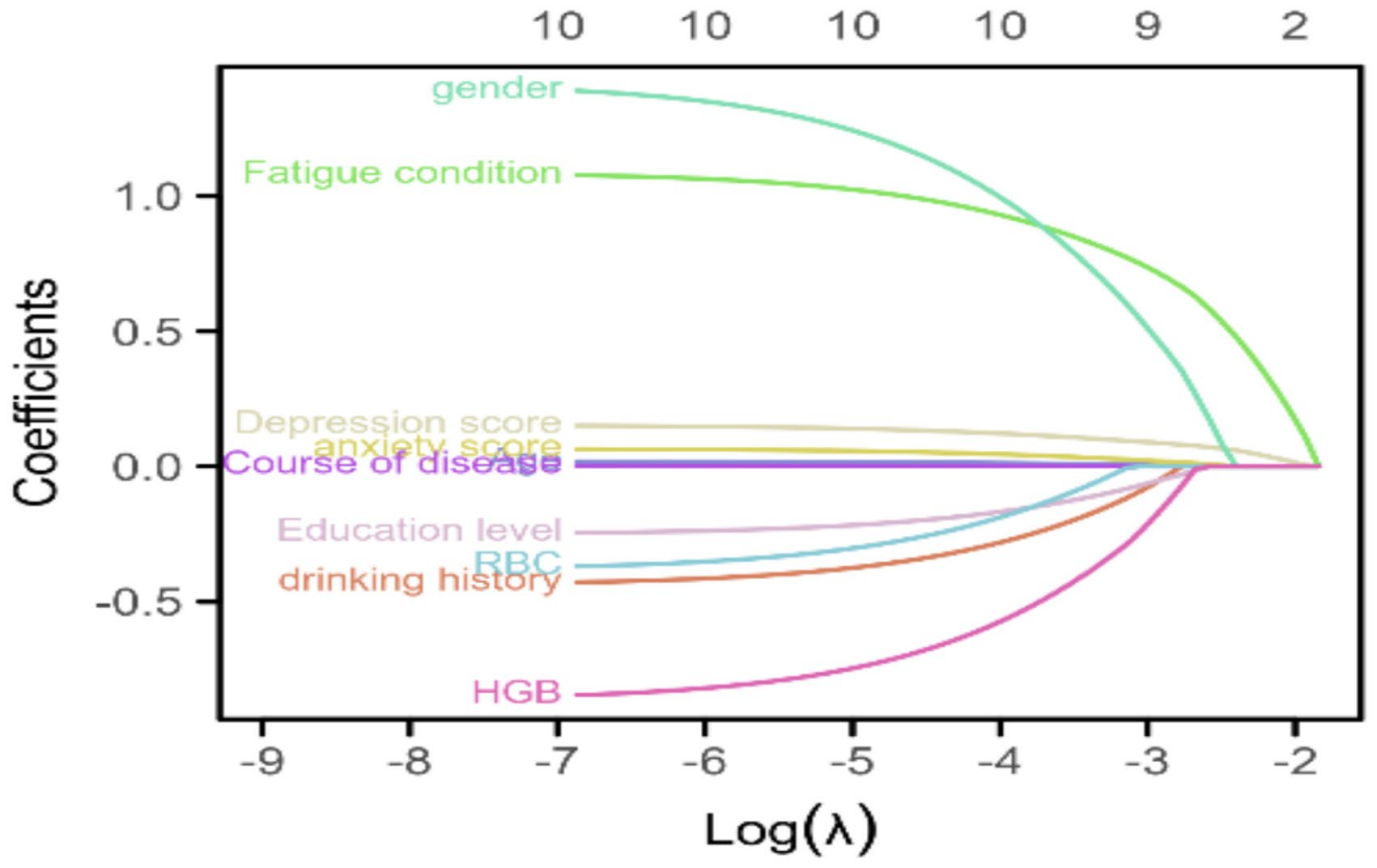
Trajectories of variables affecting sleep under LASSO regression. This figure shows the trajectories of variables that affect sleep based on the LASSO model, which gradually compresses the variable coefficients to 0 as λ changes, thereby achieving the purpose of variable selection.

**Figure 3:**
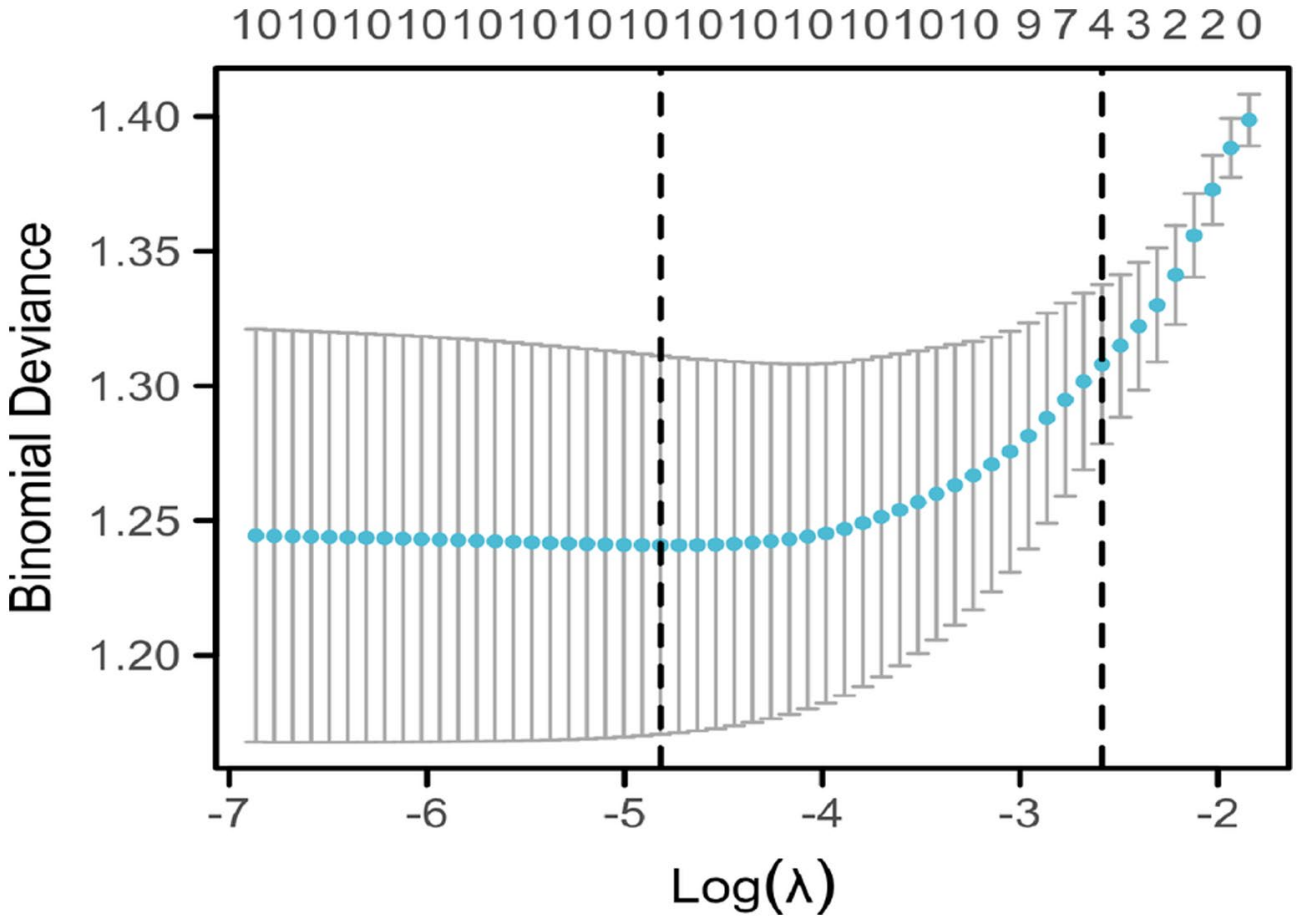
Coefficient selection of indicators affecting sleep under LASSO regression. This figure shows the relationship between log(λ) and mean squared error. The LASSO method was used for variable selection using 10-fold cross-validation. The figure shows that ten factors were further analyzed using multivariate logistic regression to construct the logistic model.

Figure 2 shows the trajectories of variables that affect sleep based on the LASSO model, which gradually compresses the variable coefficients to 0 as *λ* changes, thereby achieving the purpose of variable selection. Figure 3 shows the relationship between log(λ) and mean squared error. The LASSO method was used for variable selection using 10-fold cross-validation. The figure shows that 10 factors were further analyzed using multivariate logistic regression to construct the logistic model. The final selected variables were age, gender distribution, disease duration, education level, history of alcohol consumption, anxiety score, depression score, fatigue, RBC, and HGB (Figures 2, 3).

### 3.4 Binary logistic regression analysis

The variables selected by the LASSO model were used as the independent variables in a binary logistic regression model, and variable selection was performed using a forward LR method. Based on the results of the multivariate logistic regression analysis, gender distribution, high fatigue level, and high depression score were identified as the significant predictors of poor sleep quality (Table 4).

**Table 4:** Binary logistic regression analysis.

Variable	Standardized coefficients (β)	*p*	OR	95%CI
Gender	1.549	0.010	4.706	1.449–15.290
Fatigue	1.286	0.000	3.617	1.854–7.057
Depression score	0.188	0.001	1.207	1.078–1.352

### 3.5 Nomogram model

**Figure 4:**
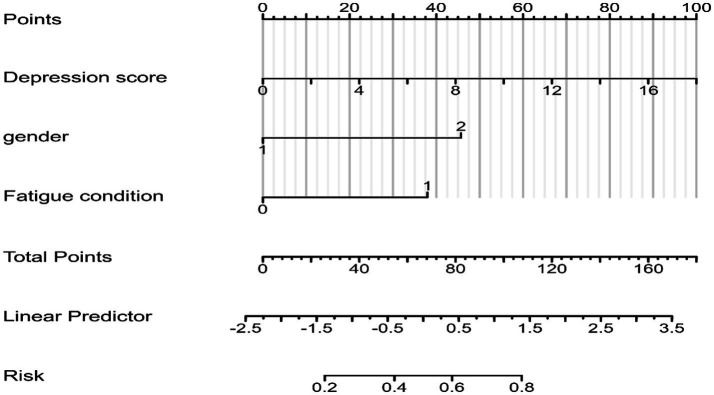
Nomogram for the risk of poor sleep quality in SLE patients.

Based on the results of the multivariate logistic regression analysis, a nomogram was constructed using the risk factors of gender, fatigue, and depression score. The left side of the model represents the individual scores corresponding to different values of each predictor variable. Each variable in the model is marked with a scale on the corresponding line segment, representing the range of values the variable can take and the length of the line segment reflects the contribution of that factor to the outcome event “Total Points” represents the total score obtained by adding up the single-item scores for each variable, and “Linear Predictor” represents the linear predicted value (Figure 4).

### 3.6 Model validation

#### 3.6.1 ROC curve analysis

**Figure 5:**
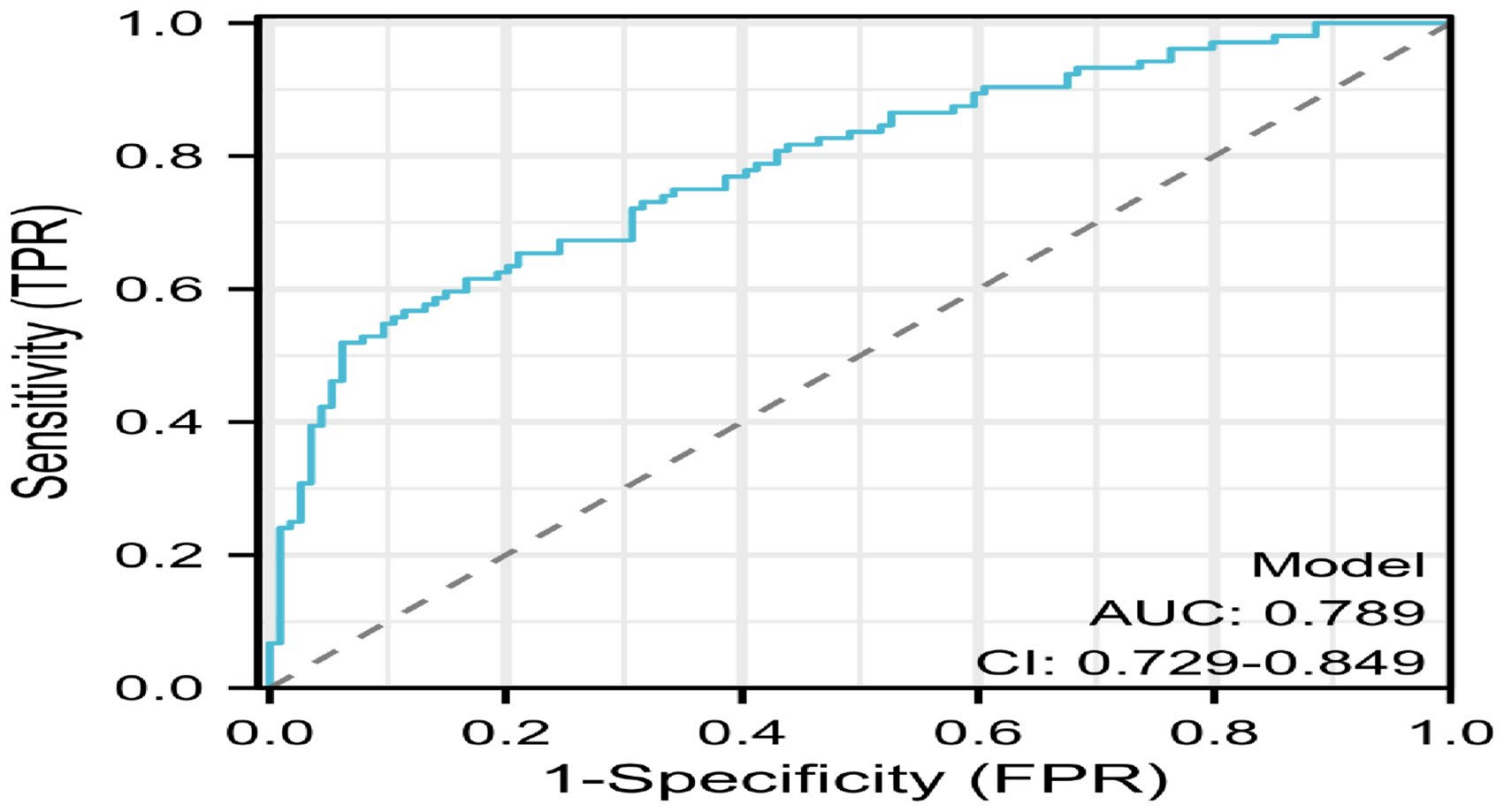
Receiver operating characteristic curve analysis of poor sleep quality and predictive probability. Receiver operating characteristic curve analysis of poor sleep quality and predictive probability. ROC curve analysis was performed to assess the predictive probability of the model for the presence or absence of poor sleep quality. The AUC was 0.789 (95% CI: 0.729–0.849), while sensitivity and specificity were 51.92% and 93.86%, respectively, indicating a good discriminatory ability of the nomogram.

ROC curve analysis was performed to assess the predictive probability of the model for the presence or absence of poor sleep quality. The AUC was 0.789 (95% CI: 0.729–0.849), while sensitivity and specificity were 51.92 and 93.86%, respectively, indicating a good discriminatory ability of the nomogram (Figure 5).

#### 3.6.2 Calibration curve analysis and calibration curve analysis

**Figure 6:**
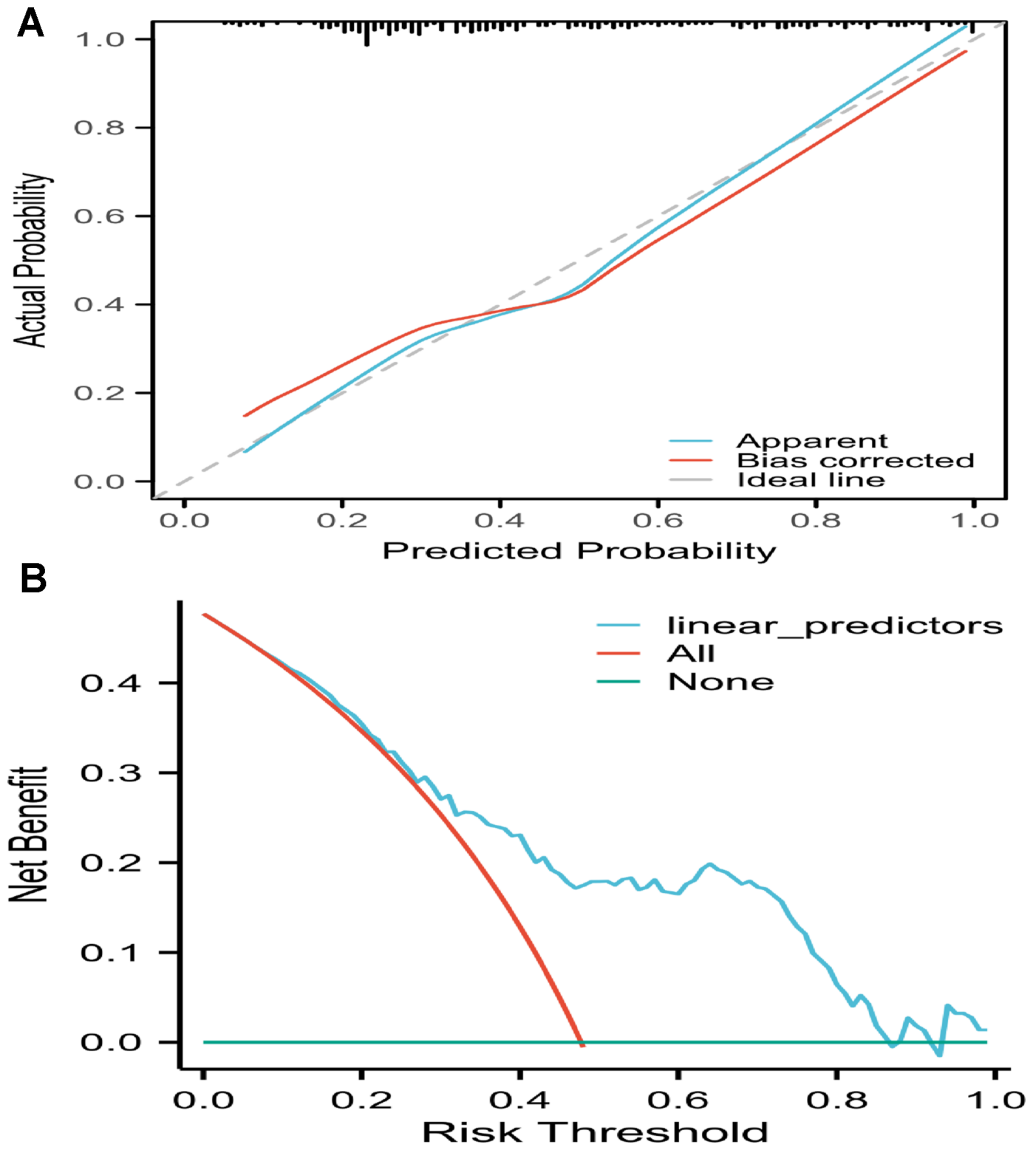
Calibration curve of the prediction model and decision curve analysis of the prediction model. Calibration curve of the prediction model and decision curve analysis of the prediction model. **(A)** The calibration curve of the prediction model was a straight line with a slope close to 1. **(B)** The decision curve analysis of the prediction model showed a threshold probability of 35%, indicating that it has good clinical application value.

After 2000 rounds of internal validation using bootstrap resampling, the concordance index (C-index) of the nomogram was found to be 0.789, which was consistent with the AUC result from the ROC analysis. The calibration curve of the prediction model was a straight line with a slope close to 1, the decision curve analysis of the prediction model showed a threshold probability of 35%, indicating that it has good clinical application value (Figure 6).

#### 3.6.3 Clinical impact curve analysis

**Figure 7:**
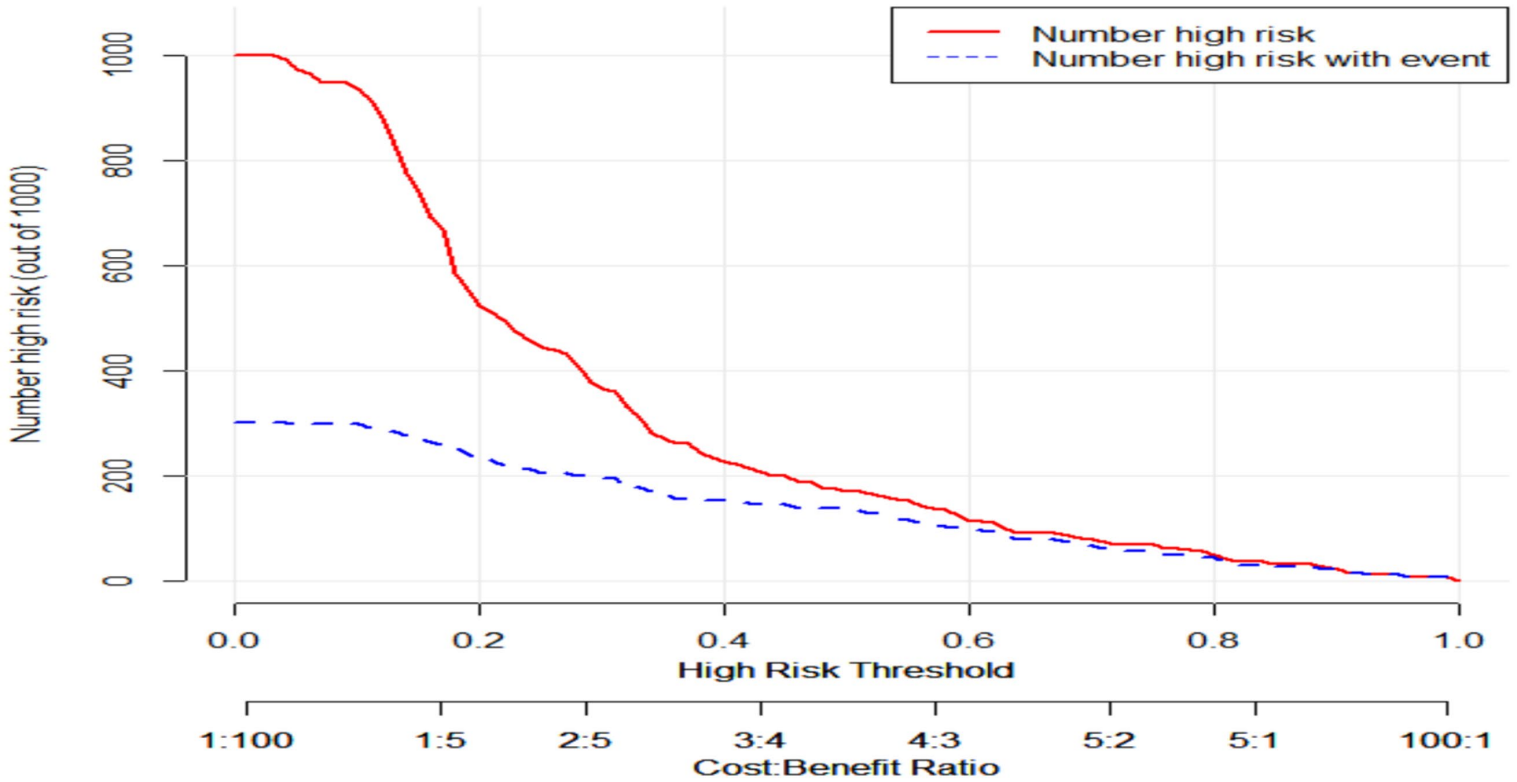
Clinical impact curve of the prediction model. Clinical impact curve of the prediction model. This figure depicts the clinical effectiveness of the predictive model. When the threshold probability exceeds 60% of the predicted score probability, the model can identify high-risk individuals.

Clinical impact curve (CIC) analysis demonstrated the clinical effectiveness of the prediction model. When the threshold probability exceeded 60% of the predicted score probability, the prediction model identified individuals at high risk of poor sleep quality, which matched well with the actual high-risk population for poor sleep quality, confirming the high clinical effectiveness of the prediction model (Figure 7).

## 4 Discussion

SLE is a common autoimmune disease that is often accompanied by poor sleep quality. Previous reports have shown that the occurrence rate of poor sleep quality in SLE ranges from 42% to over 81% (9, 28–30), which is consistent with the findings of the present study (47.7%). Sleep is a basic human heed and is crucial for both physical and mental health. When sleep quality declines, it can worsen the patient’s disease experience and have a negative impact on health and quality of life (31). Therefore, early identification of individuals with SLE and poor sleep quality and providing early intervention are particularly important. In the present study, a wide range of variables were considered to comprehensively understand the sleep situation and further explore the influencing factors of poor sleep quality in SLE patients.

Previous studies have shown that depression symptoms (32, 33) and negative emotions (34) correlate with the severity of poor sleep quality. Many researchers have suggested that anxiety (35), depression (36), and fatigue (37, 38) are factors influencing sleep quality. It has also been reported that disease activity and depression are confounding factors for poor sleep quality in SLE patients (14). The relationship between disease activity and depression has been found to strongly influence depression symptoms (39), suggesting that disease activity may act on depression, and the accumulated depressive mood may affect sleep quality.

In addition to affecting sleep quality, depression itself can exacerbate fatigue, and thus, affect sleep. Attree et al. (40) pointed out that although the pathological and physiological mechanisms of fatigue are still unclear, immune system abnormalities are clearly present. Groven et al. (41) found that patients with fatigue symptoms had higher tumor necrosis factor-alpha (TNF-*α*) plasma levels compared with healthy controls, and also that these daytime increases in inflammatory cytokines may explain the increased fatigue experienced during the day (13). Therefore, there is an apparent relationship between fatigue and poor sleep quality. Long-term sleep deprivation or poor sleep quality can increase fatigue and affect daily life and work efficiency. Fatigue can also disrupt sleep. The interaction between poor sleep quality and fatigue can form a cycle that requires intervention and adjustment to reduce potential negative impacts.

Researchers have suggested that anxiety (35) and depression (36) can influence sleep quality. This may be related to the decreased melatonin levels in patients with depression, as melatonin has a sedative effect and can help with falling asleep. More research should be conducted to provide further evidence for sleep management in patients with SLE.

Based on studies on clinical populations, 36–91% of patients report insomnia either while drinking or within several weeks of stopping (42). In a study of alcohol consumption in SLE, drinkers were more likely to feel psychologically stressed than non-drinkers (43).

A review provides a qualitative assessment of all known scientific studies on the effect of alcohol ingestion on nocturnal sleep in healthy volunteers. At all dosages, alcohol causes a reduction in sleep onset latency, a more consolidated first half of sleep, and an increase in sleep disruption in the second half of sleep (44–47). Disturbances in sleep and circadian rhythms may be important risk factors for the initiation of alcohol use and the escalation of alcohol problems (48), which is consistent with this study.

The nomogram transforms complex regression equations into a simple and visual graphical tool, which quantifies the risk of clinical diseases in individuals by integrating various risk factors (49). It is an implementable tool for risk prediction and patient management. This study included indicators specific to the population characteristics, as well as SLE symptom indicators, to preliminarily screen factors influencing the sleep quality of SLE patients using LASSO regression analysis, and multicollinearity was assessed using VIF (50). Finally, we constructed a nomogram with good predictive performance. The concordance index (C-index) of the nomogram was found to be 0.789, with the value of the C-index ranging from 0.5 to 1.0, where 0.5 indicates random chance and 1.0 indicates the model’s perfect ability to correctly predict the outcome (51). This was consistent with the AUC result from the ROC analysis, indicating that the nomogram had excellent predictive ability. The calibration curve of the prediction model was a straight line with a slope close to 1, indicating there was good consistency between the predicted risk of poor sleep quality in SLE patients and the actual risk. Decision curve analysis of the prediction model showed a threshold probability of 35%. The net benefit is useful for determining whether basing clinical decisions on a model would do more good than harm (52), indicating the high clinical utility of the model. The CIC analysis demonstrated the clinical effectiveness of this prediction model, This nomogram has good discriminatory ability, so it can be used when the physician has a high suspicion that the patient may have or develop sleep disturbance based on the clinical presentation or activity assessment of the patient; however, it is not recommended for screening of sleep quality in a wide range of patients with SLE. However, it is worth noting that the constructed nomogram for early screening of poor sleep quality in SLE patients provides a convenient and practical model with strong clinical applicability and good health, economic, and social benefits.

Although anemia may affect sleep, abnormalities in RBC and HGB are also a manifestation of SLE and reflect the changes in quality of life that the disease may bring to patients, so we did not consider it a confounding factor in the analysis. This study found the effect of sex differences in terms of sleep in patients with SLE. Women were found to be at a higher risk of sleep disorders with a risk ratio of 1.41 (95% confidence interval: 1.28–1.55) for women versus men. The risk of women experiencing insomnia was found to be significantly higher than that of men in large and high-quality studies, confirming that women are predisposed to insomnia (53, 54).

In conclusion, age, gender, disease duration, history of alcohol consumption, education level, anxiety score, depression score, fatigue, RBC, and HGB are all associated with poor sleep quality in individuals with SLE, while female sex, high fatigue, and high depression scores are independent risk factors for poor sleep quality in SLE. The nomogram constructed based on the results of both univariate and multivariate logistic regression analyses shows good predictive performance and could be of clinical significance for the prevention and intervention of poor sleep quality in high-risk SLE patients.

### 4.1 Limitations

There were some limitations to the study. First, as the study design was cross-sectional, we could not make causal inferences. Second, external validation of the prediction model was not performed, and only internal validation was conducted. This study was conducted in a single center, so there may be data biases. Although nomograms are widely used clinically, they are rarely evaluated prospectively to determine whether their use improves patient outcomes. Subsequent research should be carried out in a prospective study. In future research, incorporating objective tools such as actigraphy or polysomnography could provide a comprehensive assessment of sleep disturbances in SLE patients. In addition, further research is needed to understand how sleep can be improved to foster its regulation of inflammation and immunosupportive functions.

## 5 Conclusion

In summary, the model demonstrated favorable accuracy, calibration, and practicability, the clinical value of the nomogram lies in transforming complex sleep into an actionable predictive tool, that is especially suitable for early screening and stratified management in preventive scenarios. The nomogram can be iteratively updated by regularly incorporating new data into it. Future directions for research include the development of dynamic models, exploration of causal mechanisms, and achievement of closed-loop management from symptom assessment to precise intervention.

## Data Availability

The original contributions presented in the study are included in the article/supplementary material, further inquiries can be directed to the corresponding author.

## References

[1] Barnett R. Systemic lupus erythematosus. Lancet. (2016) 387:1711. doi: 10.1016/s0140-6736(16)30266-5 27116268

[2] Rees F Doherty M Grainge MJ Lanyon P Zhang W. The worldwide incidence and prevalence of systemic lupus erythematosus: a systematic review of epidemiological studies. Rheumatology. (2017) 56:1945–61. doi: 10.1093/rheumatology/kex260, PMID: 28968809

[3] Tektonidou MG Lewandowski LB Hu J Dasgupta A Ward MM. Survival in adults and children with systemic lupus erythematosus: a systematic review and Bayesian meta-analysis of studies from 1950 to 2016. Ann Rheum Dis. (2017) 76:2009–16. doi: 10.1136/annrheumdis-2017-211663, PMID: 28794077

[4] Giacomelli R Afeltra A Alunno A Baldini C Bartoloni-Bocci E Berardicurti O. International consensus: what else can we do to improve diagnosis and therapeutic strategies in patients affected by autoimmune rheumatic diseases (rheumatoid arthritis, spondyloarthritides, systemic sclerosis, systemic lupus erythematosus, antiphospholipid syndrome and Sjogren's syndrome)?: the unmet needs and the clinical grey zone in autoimmune disease management. Autoimmun Rev. (2017) 16:911–24. doi: 10.1016/j.autrev.2017.07.012, PMID: 28705780

[5] Mahieu MA Ahn GE Chmiel JS Dunlop DD Helenowski IB Semanik P. Fatigue, patient reported outcomes, and objective measurement of physical activity in systemic lupus erythematosus. Lupus. (2016) 25:1190–9. doi: 10.1177/0961203316631632, PMID: 26869353 PMC4980272

[6] Cervilla O Miró E Martínez MP Sánchez AI Sabio JM Prados G. Sleep quality and clinical and psychological manifestations in women with mild systemic lupus erythematosus activity compared to women with fibromyalgia: a preliminary study. Mod Rheumatol. (2020) 30:1016–24. doi: 10.1080/14397595.2019.1679973, PMID: 31599659

[7] Madsen LT. Cancer prediction nomograms for advanced practitioners in oncology. J Adv Pract Oncol. (2014) 5:380–2. doi: 10.6004/jadpro.2014.5.5.9, PMID: 26114019 PMC4457177

[8] Ruiz-Irastorza G Khamashta MA Castellino G Hughes GRV. Systemic lupus erythematosus. Lancet. (2001) 357:1027–32. doi: 10.1016/s0140-6736(00)04239-2 11293608

[9] Inoue M Shiozawa K Yoshihara R Yamane T Shima Y Hirano T. Predictors of poor sleep quality in patients with systemic lupus erythematosus. Clin Rheumatol. (2017) 36:1053–62. doi: 10.1007/s10067-017-3545-5, PMID: 28138857

[10] Moraleda V Prados G Martínez MP Sánchez AI Sabio JM Miró E. Sleep quality, clinical and psychological manifestations in women with systemic lupus erythematosus. Int J Rheum Dis. (2017) 20:1541–50. doi: 10.1111/1756-185x.13081, PMID: 28425178

[11] Abad VC Sarinas PSA Guilleminault C. Sleep and rheumatologic disorders. Sleep Med Rev. (2008) 12:211–28. doi: 10.1016/j.smrv.2007.09.001 18486034

[12] Hinze AM Chu P Sen DP Al-Hammadi N Ju Y-ES Kim AH. 55 poor sleep quality assessed subjectively associated with worsening SLE disease activity. Lupus Sci Med. (2019) 6:A40–1. doi: 10.1136/lupus-2019-lsm

[13] Besedovsky L Lange T Haack M. The sleep-immune crosstalk in health and disease. Physiol Rev. (2019) 99:1325–80. doi: 10.1152/physrev.00010.2018, PMID: 30920354 PMC6689741

[14] Mirbagher L Gholamrezaei A Hosseini N Sayed BZ. Sleep quality in women with systemic lupus erythematosus: contributing factors and effects on health-related quality of life. Int J Rheum Dis. (2016) 19:305–11. doi: 10.1111/1756-185x.12418 24910903

[15] Moons KGM Royston P Vergouwe Y Grobbee DE Altman DG. Prognosis and prognostic research: what, why, and how? BMJ. (2009) 338:b375. doi: 10.1136/bmj.b375, PMID: 19237405

[16] Liu C Zhao W Xie J Lin H. Development and validation of a radiomics-based nomogram for predicting a major pathological response to neoadjuvant immunochemotherapy for patients with potentially resectable non-small cell lung cancer. Front Immunol. (2023) 14:1115291. doi: 10.3389/fimmu.2023.1115291, PMID: 36875128 PMC9978193

[17] Hu C. Nomogram: a better method for evaluating MVD risk. Int J Cardiol. (2024) 411:132283. doi: 10.1016/j.ijcard.2024.132283, PMID: 38906422

[18] Petri M Orbai A-M Alarcón GS Gordon C Merrill JT Fortin PR. Derivation and validation of the systemic lupus international collaborating clinics classification criteria for systemic lupus erythematosus. Arthritis Rheum. (2012) 64:2677–86. doi: 10.1002/art.34473, PMID: 22553077 PMC3409311

[19] Wu L Shi P-L Tao S-S Tao J-H Wu G-C. Decreased sleep quality in patients with systemic lupus erythematosus: a meta-analysis. Clin Rheumatol. (2021) 40:913–22. doi: 10.1007/s10067-020-05300-3 32748069

[20] Buysse DJ Reynolds CF Monk TH Berman SR Kupfer DJ. The Pittsburgh sleep quality index: a new instrument for psychiatric practice and research. Psychiatry Res. (1989) 28:193–213. 2748771 10.1016/0165-1781(89)90047-4

[21] Liu X Tang M Hu L Wang A Wu H Zhao G. Reliability and validity of the Pittsburgh sleep quality index. Chin J Psychiatry. (1996) 29:103–7.

[22] Tibshirani R. Regression shrinkage and selection via the lasso. J R Stat Soc Ser B Stat Methodol. (1996) 58:267–88.

[23] Fontanarosa JB Dai Y. Using LASSO regression to detect predictive aggregate effects in genetic studies. BMC Proc. (2011) 5:S69. doi: 10.1186/1753-6561-5-S9-S69 22373537 PMC3287908

[24] Tripepi G Jager KJ Dekker FW Zoccali C. Diagnostic methods 2: receiver operating characteristic (ROC) curves. Kidney Int. (2009) 76:252–6. doi: 10.1038/ki.2009.171, PMID: 19455194

[25] Iasonos A Schrag D Raj GV Panageas KS. How to build and interpret a nomogram for cancer prognosis. J Clin Oncol. (2008) 26:1364–70. doi: 10.1200/jco.2007.12.9791 18323559

[26] Steyerberg EW Borsboom GJJM van Houwelingen HC Eijkemans MJC Habbema JDF. Validation and updating of predictive logistic regression models: a study on sample size and shrinkage. Stat Med. (2004) 23:2567–86. doi: 10.1002/sim.1844, PMID: 15287085

[27] Concato J Peduzzi P Holford TR Feinstein AR. Importance of events per independent variable in proportional hazards analysis I. Background, goals, and general strategy. J Clin Epidemiol. (1995) 48:1495–501. 8543963 10.1016/0895-4356(95)00510-2

[28] Liu D Zuo X Zeng F Xu Q Liu H Li Y. Associated factors on sleep disorders in systemic lupus erythematosus patients. Chin J Rheumatol. (2018) 22:309–13. doi: 10.3760/cma.j.issn.1007-7480.2018.05.005

[29] Gadie A Shafto M Leng Y Kievit RA Cam CAN. How are age-related differences in sleep quality associated with health outcomes? An epidemiological investigation in a UK cohort of 2406 adults. BMJ Open. (2017) 7:e014920. doi: 10.1136/bmjopen-2016-014920, PMID: 28760786 PMC5642766

[30] Durcan G Sahin S Koyuncu Z Yıldız M Hacıvelioglu E Haslak F. An evaluation of sleep habits and childhood-onset systemic lupus erythematosus. Clin Rheumatol. (2022) 41:2831–7. doi: 10.1007/s10067-022-06225-9, PMID: 35639260

[31] Choi MY Malspeis S Sparks JA Cui J Yoshida K Costenbader KH. Association of sleep deprivation and the risk of developing systemic lupus erythematosus among women. Arthritis Care Res. (2023) 75:1206–12. doi: 10.1002/acr.25017, PMID: 36094865 PMC10008454

[32] Palagini L Tani C Bruno RM Gemignani A Mauri M Bombardieri S. Poor sleep quality in systemic lupus erythematosus: does it depend on depressive symptoms? Lupus. (2014) 23:1350–7. doi: 10.1177/0961203314540762, PMID: 24944187

[33] Lillis TA Tirone V Gandhi N Weinberg S Nika A Sequeira W. Sleep disturbance and depression symptoms mediate relationship between pain and cognitive dysfunction in lupus. Arthritis Care Res. (2019) 71:406–12. doi: 10.1002/acr.23593, PMID: 29726637

[34] Vina ER Green SL Trivedi T Kwoh CK. Correlates of sleep abnormalities in systemic lupus: a cross-sectional survey in an urban, academic center. J Clin Rheumatol. (2013) 19:7–13. doi: 10.1097/rhu.0b013e31827cd20d, PMID: 23319017

[35] Gong L Cheng S Gao G Zhang F. Analysis of the sleep quality of patients with systemic lupus erythematosus and its influencing factors. Chin J Mod Nurs. (2018) 24:393–7. doi: 10.3760/cma.j.issn.1674-2907.2018.04.005

[36] Mesquita RC Souza LNC Bruin PFC Carvalho RR Medeiros MMC Rocha FAC. Sleep disturbances and prevalence of depression in systemic lupus erythematosus patients receiving intravenous cyclophosphamide. Rev Bras Reumatol. (2007) 47:396–400. doi: 10.1590/s0482-50042007000600002

[37] Du X Zhuang Y Chen H Shen B. Fatigue in patients with systemic lupus erythematosus: a review. Nurs J Chin PLA. (2019) 36:67–70.

[38] Tench CM McCurdie I White PD D'Cruz DP. The prevalence and associations of fatigue in systemic lupus erythematosus. Rheumatology. (2000) 39:1249–54. doi: 10.1093/rheumatology/39.11.1249, PMID: 11085805

[39] Figueiredo-Braga M Cornaby C Cortez A Bernardes M Terroso G Figueiredo M. Influence of biological therapeutics, cytokines, and disease activity on depression in rheumatoid arthritis. J Immunol Res. (2018) 2018:5954897. doi: 10.1155/2018/5954897, PMID: 30148175 PMC6083532

[40] Attree EA Arroll MA Dancey CP Griffith C Bansal AS. Psychosocial factors involved in memory and cognitive failures in people with myalgic encephalomyelitis/chronic fatigue syndrome. Psychol Res Behav Manag. (2014) 7:67–76. doi: 10.2147/PRBM.S50645, PMID: 24596470 PMC3940708

[41] Groven N Fors EA Iversen VC White LR Reitan SK. Association between cytokines and psychiatric symptoms in chronic fatigue syndrome and healthy controls. Nord J Psychiatry. (2018) 72:556–60. doi: 10.1080/08039488.2018.1493747, PMID: 30063870

[42] Brower KJ. Insomnia, alcoholism and relapse. Sleep Med Rev. (2003) 7:523–39. doi: 10.1016/s1087-0792(03)90005-0 15018094

[43] Washio M Horiuchi T Kiyohara C Kodama H Tada Y Asami T. Smoking, drinking, sleeping habits, and other lifestyle factors and the risk of systemic lupus erythematosus in Japanese females: findings from the KYSS study. Mod Rheumatol. (2006) 16:143–50. doi: 10.1007/s10165-006-0474-6, PMID: 16767552

[44] Ebrahim IO Shapiro CM Williams AJ Fenwick PB. Alcohol and sleep I: effects on normal sleep. Alcohol Clin Exp Res. (2013) 37:539–49. doi: 10.1111/acer.12006, PMID: 23347102

[45] Hasler BP Pedersen SL. Sleep and circadian risk factors for alcohol problems: a brief overview and proposed mechanisms. Curr Opin Psychol. (2020) 34:57–62. doi: 10.1016/j.copsyc.2019.09.005, PMID: 31629218 PMC7082179

[46] Irwin M Rinetti G Redwine L Motivala S Dang J Ehlers C. Nocturnal proinflammatory cytokine-associated sleep disturbances in abstinent African American alcoholics. Brain Behav Immun. (2004) 18:349–60. doi: 10.1016/j.bbi.2004.02.001, PMID: 15157952

[47] Gardiner C Weakley J Burke LM Roach GD Sargent C Maniar N. The effect of alcohol on subsequent sleep in healthy adults: a systematic review and meta-analysis. Sleep Med Rev. (2025) 80:102030. doi: 10.1016/j.smrv.2024.102030, PMID: 39631226

[48] Redwine L Dang J Hall M Irwin M. Disordered sleep, nocturnal cytokines, and immunity in alcoholics. Psychosom Med. (2003) 65:75–85. doi: 10.1097/01.psy.0000038943.33335.d2, PMID: 12554818

[49] Balachandran VP Gonen M Smith JJ DeMatteo RP. Nomograms in oncology: more than meets the eye. Lancet Oncol. (2015) 16:e173–80. doi: 10.1016/S1470-2045(14)71116-7, PMID: 25846097 PMC4465353

[50] Marcoulides KM Raykov T. Evaluation of variance inflation factors in regression models using latent variable modeling methods. Educ Psychol Meas. (2019) 79:874–82. doi: 10.1177/0013164418817803 31488917 PMC6713981

[51] Harrell FE Jr. Regression modeling strategies: With applications to linear models, logistic regression, and survival analysis. New York: Springer (2001). 600 p.

[52] Vickers AJ Van Calster B Steyerberg EW. Net benefit approaches to the evaluation of prediction models, molecular markers, and diagnostic tests. BMJ. (2016) 352:i6. doi: 10.1136/bmj.i6, PMID: 26810254 PMC4724785

[53] Zhang B Wing Y-K. Sex differences in insomnia: a meta-analysis. Sleep. (2006) 29:85–93. doi: 10.1093/sleep/29.1.85, PMID: 16453985

[54] Lok R Qian J Chellappa SL. Sex differences in sleep, circadian rhythms, and metabolism: implications for precision medicine. Sleep Med Rev. (2024) 75:101926. doi: 10.1016/j.smrv.2024.101926, PMID: 38564856

[55] Gladman DD IbaÑez D Urowitz MB. Systemic lupus erythematosus disease activity index 2000. J Rheumatol. (2002) 29:288–91. PMID: 11838846

[56] Jesus D Matos A Henriques C Zen M Larosa M Iaccarino L. Derivation and validation of the SLE disease activity score (SLE-DAS): a new SLE continuous measure with high sensitivity for changes in disease activity. Ann Rheum Dis. (2019) 78:365–71. doi: 10.1136/annrheumdis-2018-214502, PMID: 30626657

[57] Zigmond AS Snaith RP. The hospital anxiety and depression scale. Acta Psychiatr Scand. (1983) 67:361–70. 6880820 10.1111/j.1600-0447.1983.tb09716.x

[58] Lorentzen K Danielsen MA Kay SD Voss A. Validation of the fatigue severity scale in Danish patients with systemic lupus erythematosus. Dan Med J. (2014) 61:A4808 24814589

[59] Yang Y Zhang H. Introduction to the dietary guidelines for Chinese residents (2016). Acta Nutr Sin. (2016) 38:209–17. doi: 10.13325/j.cnki.acta.nutr.sin.2016.03.002

[60] Barbacki A Petri M Aviña-Zubieta A Alarcón GS Bernatsky S. Fatigue measurements in systemic lupus erythematosus. J Rheumatol. (2019) 46:1470–7. doi: 10.3899/jrheum.180831, PMID: 30709953

